# Deformation Behaviors and Energy Absorption of Composite Re-Entrant Honeycomb Cylindrical Shells under Axial Load

**DOI:** 10.3390/ma14237129

**Published:** 2021-11-23

**Authors:** Nanfang Ma, Qingtian Deng, Xinbo Li

**Affiliations:** Department of Engineering Mechanics, School of Science, Chang’an University, Xi’an 710064, China; 17673816018@163.com (N.M.); sinboy1016@chd.edu.cn (X.L.)

**Keywords:** re-entrant honeycomb, composite materials, cylindrical shell, energy absorption

## Abstract

Composite materials and re-entrant honeycomb structures have superior mechanical performance in energy absorption capacity. Inspired by laminate composite layers, single-layer re-entrant honeycomb cylindrical shells (RHCSs) with different orientations were established, and composite RHCSs were proposed by combining the single-layer RHCSs with different orientations. The deformation behaviors of single layer RHCSs under quasi-static compression were studied by experimentation, and single-layer RHCSs with varying orientations did not show negative Poisson’s ratio effects. The energy absorption capacity of single-layer and composite RHCSs was researched using simulation. To analyze the energy absorption capacity, we determined the plateau stress, the mean force and specific energy absorption of single-layer and multi-layer composite RHCSs under different impact velocities; the following conclusions were obtained: compared with the single-layer RHCSs, the multi-layer composite RHCSs, which had the same size, the energy absorption capacity improved significantly under the same impact velocities. The energy absorption capacity of the multi-layer composite RHCSs improved with increasing number of layers under low velocity.

## 1. Introduction

Lattice cylindrical shells such as re-entrant honeycomb [1], phase-transforming [2], rigid joint rotation [3], and hierarchical structures [4] have gained attention because of their excellent mechanical properties such as large shear resistance [5] and enhancement of fracture toughness [6], which have been widely used in the aerospace, automotive, and other engineering fields [7]. Wang et al. [8] analyzed the Voronoi cylindrical shell structure and systematically examined the crushing behavior of such honeycomb cylindrical structures by considering the cell irregularity, the relative density, and the density-graded properties. Gao et al. [9,10,11] theoretically and experimentally studied the mechanical properties of cylindrical auxetic double-arrowed honeycomb. Chiral-type auxetic cylindrical shells also show good mechanical performance under axial compression [12,13,14,15,16,17].

Compared with single-layer cylindrical shells, the energy absorption capacity [18,19,20,21] and deformation behaviors [22,23,24,25,26] of sandwich honeycomb cylindrical shells have been attracting increased attention in recent years. Lee et al. [27] produced re-entrant cylindrical tubes by 3D printing and studied the effect of the auxetic structure under low velocity. Guo et al. [28] investigated the impact performance of re-entrant honeycomb cylindrical shells under axial force and the results showed that the sandwich cylindrical shell had the best performance in terms of specific energy absorption. Chen et al. [29] theoretically and numerically studied the dynamic behavior of sandwich cylindrical shells with honeycomb configurations subjected to constant velocity impact. Additionally, the fiber composite cylindrical shells [30,31,32] showed outstanding energy absorption capacity. Bisagni [33] investigated the strength capacity in the post-buckling range of three composite cylindrical shells under axial compression. Ochelski and Gotowicki [34] analyzed the influence of the fiber reinforcement type, structure type, geometry and shape of specimens, and orientation of fibers in a layer and stacking sequence of layers on energy absorption capability. Hull [35] proved that the composite tubes consisting of only one orientation of layers do not possess a large energy absorption ability. Different orientation of fibers in a layer will improve energy absorption capacity.

To obtain re-entrant honeycomb cylindrical shells with higher energy absorption capacity, composite re-entrant honeycomb cylindrical shells (RHCSs) are proposed in this paper. RHCSs are different from sandwich re-entrant honeycomb cylindrical shells [36,37,38]. The method of constructing composite RHCSs is the same as for laminate composite materials. Single-layer RHCSs are similar to the fiber layer in laminate composite layers and the deformation modes of single-layer RHCSs were studied. The multi-layer composite RHCSs consisted of different orientations of single-layer RHCSs. The energy absorption and deformation behaviors of the composite RHCSs were studied using a numerical method. In addition, the influence of different layers on composite RHCSs was studied.

## 2. Models

### 2.1. Models Design

**Figure 1:**
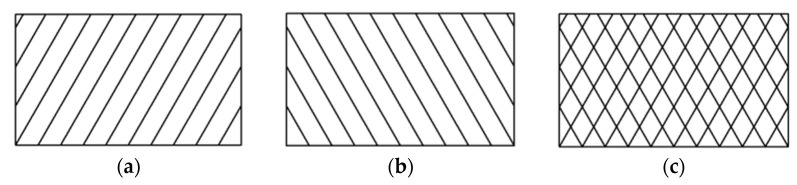
The schematic of laminate composite layers: (**a**) *θ*° layer; (**b**) −*θ*° layer; (**c**) *θ*°/−*θ*° laminate composite layer.

**Figure 2:**
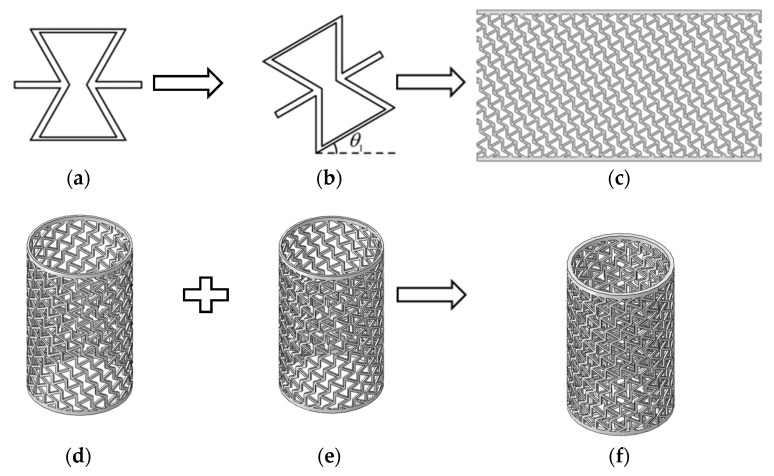
The process of constructing [*θ*_1_/−*θ*_1_] composite RHCS: (**a**) 0° re-entrant honeycomb cell; (**b**) *θ*_1_ re-entrant honeycomb cell; (**c**) *θ*_1_ re-entrant honeycomb layer; (**d**) *θ*_1_ RHCS; (**e**) −*θ*_1_ RHCS; (**f**) [*θ*_1_/−*θ*_1_] composite RHCS.

A schematic of the laminate composite layers is shown in Figure 1. Laminate composite layers have better energy absorption than single layers. Inspired by laminate composite layers, composite re-entrant honeycomb cylindrical shells (RHCSs) can be constructed in the same way. The *θ*_1_ re-entrant honeycomb layer was obtained by rotating the 0° re-entrant honeycomb layer, and the *θ*_1_ RHCS was constructed by rolling the *θ*_1_ re-entrant honeycomb layer. The [*θ*_1_/−*θ*_1_] composite RHCSs was fabricated by connecting *θ*_1_ RHCS and −*θ*_1_ RHCS. To research structures with the same mass and volume, the *θ*_1_ RHCS used to fabricate [*θ*_1_/−*θ*_1_] composite RHCS was half the width compared with single-layer *θ*_1_ RHCS. The process of constructing [*θ*_1_/−*θ*_1_] composite RHCS is shown in Figure 2.

**Figure 3:**
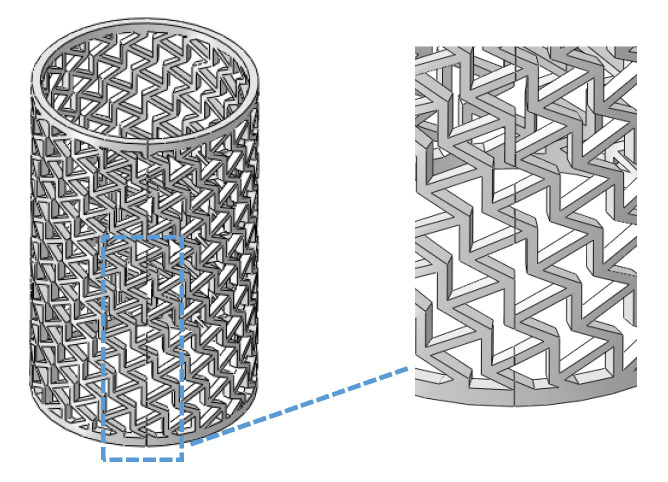
The joints of *θ*_1_ RHCSs.

**Figure 4:**
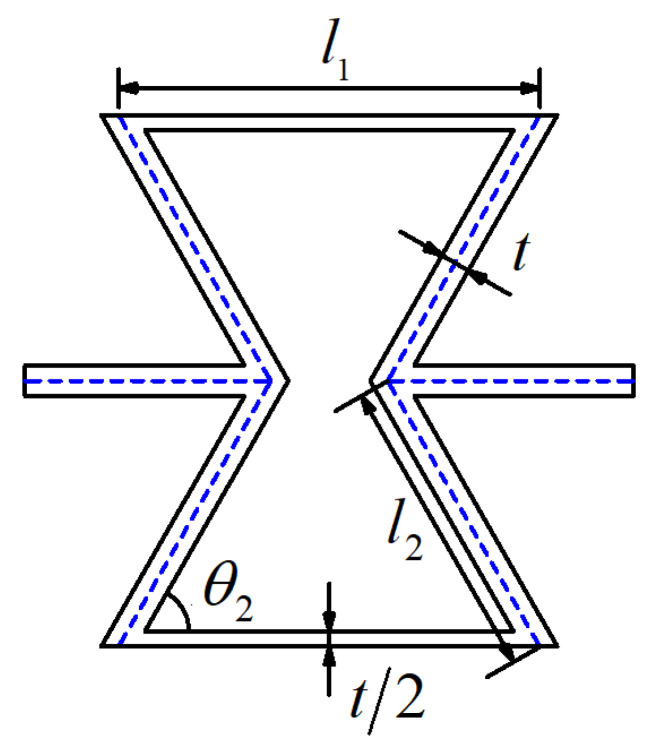
Geometrical configuration of a re-entrant honeycomb cell.

To ensure the stability of the structure and avoid structural defects, the honeycomb cylindrical shells need to be joined to form a complete cell, as shown in Figure 3. Therefore, the geometric expression of re-entrant honeycomb cell was deduced, and can be found in Appendix A. The geometric configuration of a re-entrant honeycomb cell is shown in Figure 4.

The geometric dimensions of re-entrant honeycomb cell are $l_{1}=15\;\mathrm{mm}$, $l_{2}=10\;\mathrm{mm}$, an angle of $\theta_{2}=60^{{}^{\circ}}$, and a cell wall thickness t=2 mm. For honeycomb structures, the deformation mode and energy absorption performance are highly related to two important factors: relative density and impact velocity. Based on the previous theoretical analysis [39], the relative densities of re-entrant honeycomb can be calculated as

(1) $${\bar{\rho }}_{RH}=\frac{\rho_{RH}^{*}}{\rho_{s}}=\frac{1}{2}\cdot \frac{t}{l_{2}}\cdot \frac{\left(l_{1}/l_{2}+2\right)}{(l_{1}/l_{2}-\cos\theta_{2})\sin\theta_{2}}$$

where ${\bar{\rho }}_{RH}$ is the relative density of hexagon and re-entrant honeycomb, $\rho_{RH}^{*}$ is the densities of hexagon and re-entrant honeycomb, and $\rho_{s}$ is the density of the bulk material.

**Figure 5:**
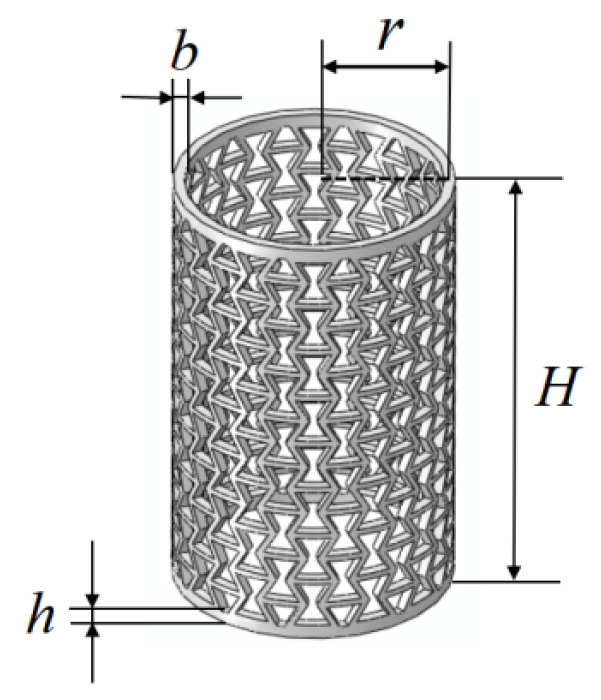
Geometrical configuration of RHCS.

The geometrical configuration of RHCS is shown in Figure 5. Based on the geometric parameters of RHCSs, the values of *θ*_1_ were chosen as 0°, ±30°, ±60°, and 90° to build 0°, ±30°, ±60°, and 90° single-layer RHCS, respectively. The height of the top and bottom base of RHCS are h = 4 mm, the overall height of RHCS is H = 146.56 mm, and the radial width of RHCS is b = 3.6 mm. The geometrical dimensions of RHCSs are listed in Table 1.

### 2.2. Fabrication of Models

**Figure 6:**
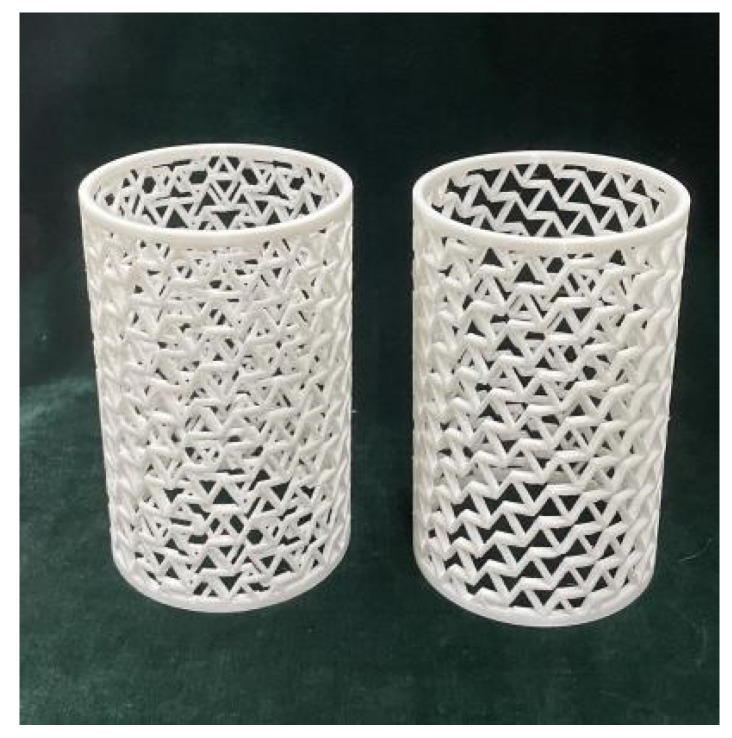
60° RHCS and [60°/−60°] RHCS.

A 3D printer was used to fabricate the dog-bones and RHCS structures, in which the printing feedstock is polylactic acid (PLA). The single-layer and composite of RHCSs were printed, as shown in Figure 6.

## 3. Method

### 3.1. Experiment and Simulation

**Figure 7:**
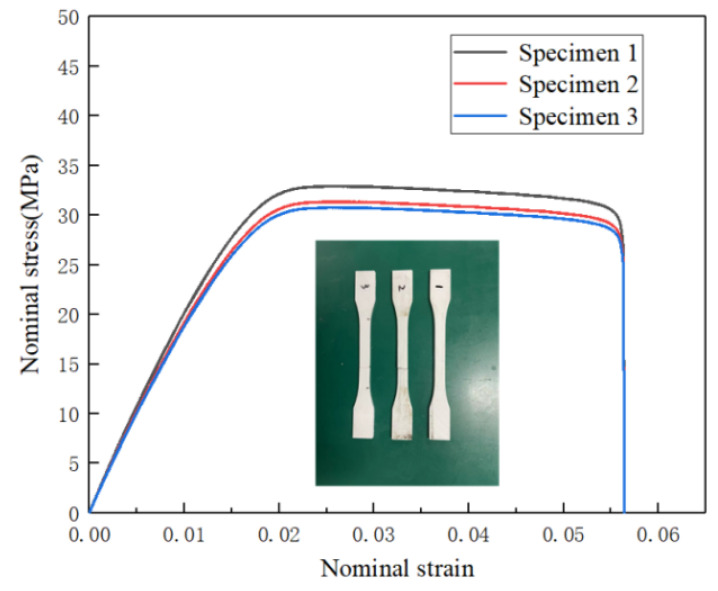
The nominal stress-strain curves of the PLA material.

The mechanical properties of the PLA can be obtained by tensile tests of dog-bone-shaped samples. As shown in Figure 7, the Young’s modulus (E) of the PLA material 1.57 GPa and yield stress (σ_ys_) is 30 MPa.

Quasi-static compression tests were performed to investigate the mechanical properties of cylindrical structures using a testing machine. The quasi-static uniaxial compression tests were conducted with a loading speed of 1 mm/min.

**Figure 8:**
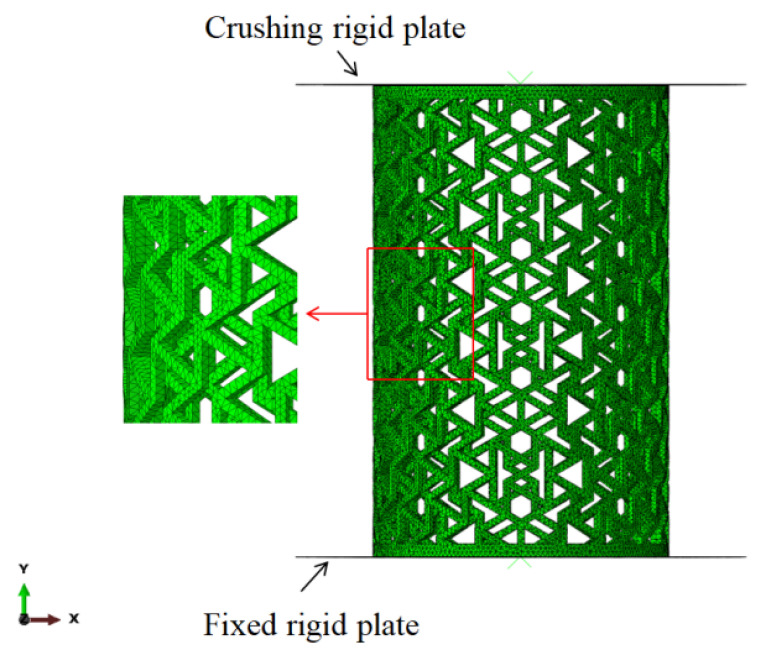
Finite element model and boundary conditions of [30°/−30°] RHCS.

**Figure 9:**
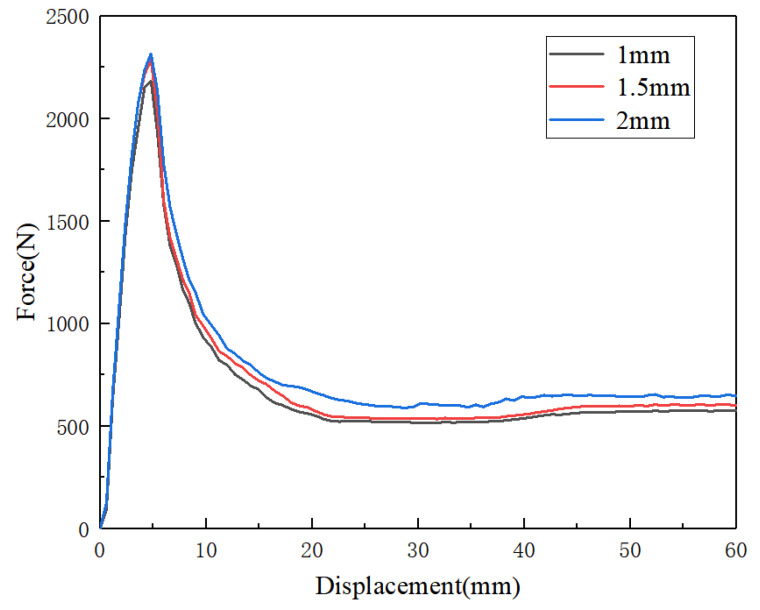
The load–displacement curves of [30°/−30°] RHCS when the velocity was 1 m/s.

Finite element software ABAQUS/Explicit (2020) was used to simulate the quasi-static compression process of honeycomb cylindrical shells. The experimental data for the PLA material were fitted in ABAQUS. The boundary condition is shown in Figure 8. The bottom plate was fixed and the top plate was given a vertical constant velocity of 1 m/s. The cylindrical shells were contacted with plates and a surface-to-surface contact was employed with a fraction coefficient of 0.3. General contact was adopted to simulate the complex mutual contact during compression. The friction coefficient of the tangential behavior was 0.15, and the Hard contact was selected for the normal behavior. A 10-node modified quadratic tetrahedron, namely the C3D10M element, was used to mesh the cylindrical shells. A mesh sensitivity analysis was carried out to guarantee that the simulation results were not mesh-dependent; the force-displacement curves of three kinds of mesh size are shown in Figure 9. A mesh size of 1.5 mm was determined to be optimal, which balanced the numerical stability, accuracy, and computational efficiency.

### 3.2. Validation of Simulation Results

**Figure 10:**
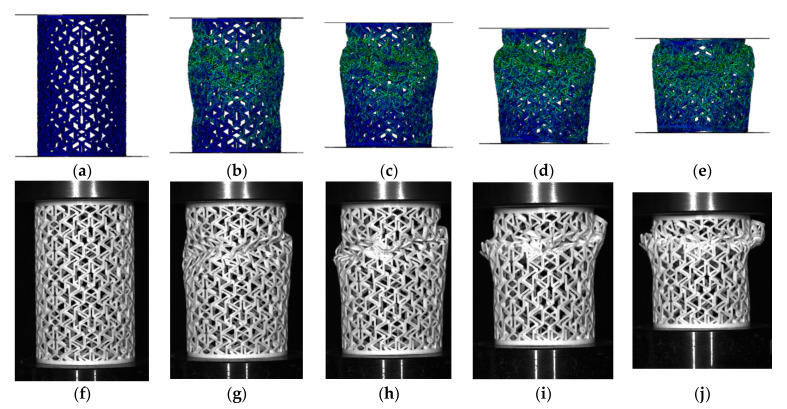
Comparison of the simulated and experimental deformation behaviors of [30°/−30°] RHCS: (**a**) 0 mm, (**b**) 8 mm, (**c**) 15 mm, (**d**) 27 mm, and (**e**) 50 mm during simulation; (**f**) 0 mm, (**g**) 8 mm, (**h**) 15 mm, (**i**) 27 mm, and (**j**) 50 mm for the experiments.

**Figure 11:**
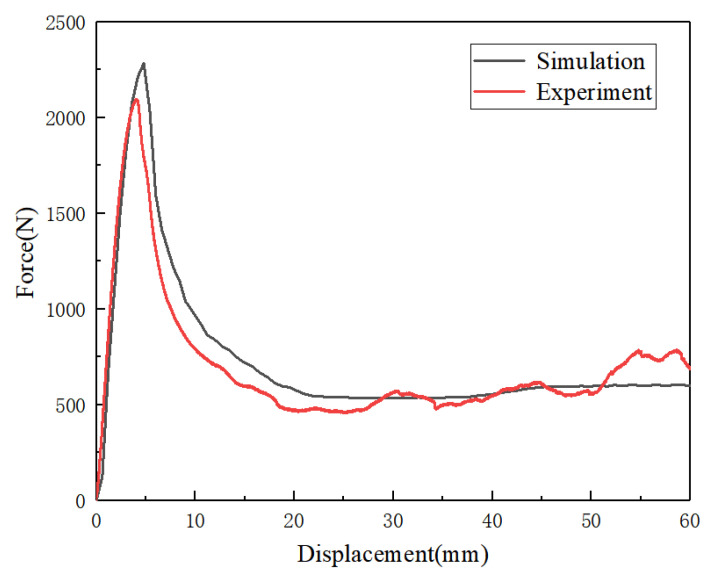
Comparison of simulated and experimental load–displacement curves of [30°/−30°] RHCS.

As shown in Figure 10 and Figure 11, the simulation and the experimental results were compared, including the load–displacement curves and the deformation process of the structures. Both the deformation modes and the load–displacement curves are in good agreement by comparing the experimental with the simulation results. This confirms that the setting of the finite element in this case was correct and effective.

## 4. Results and Discussions

### 4.1. The Theory of Energy Absorption

There are three parameters used to characterize the energy absorption of honeycomb cylindrical shells: the specific energy absorption (SEA), the mean crushing force (MCF), and the non-dimension equivalent plateau stress $EA/(L*A*\sigma_{y})$ [40].

(2) $$SEA(L)=\frac{EA(L)}{M}$$

where M is the mass of structure and EA(L) represents the total energy absorption during the crushing process that can be obtained by integrating the instantaneous crushing force. L represents the crush deformation displacement, which is set to be the axial deformation displacement corresponding to 80% strain of the structures [8].

(3) $$EA(L)=\int_{0}^{L}F(x)dx$$

The mean crushing force (MCF) represents the average force during the crushing process, which is expressed as

(4) $$MCF=\frac{EA(L)}{L}$$

The equivalent plateau stress $EA(L)/(L*A*\sigma_{y})$ can reveal the resistance strength of the structure, where A is the area of the top side of the cylindrical shell. To eliminate the influence of the cell material yield stress, the equivalent plateau stress is divided by $\sigma_{y}$ to obtain the non-dimension equivalent plateau stress $EA(L)/(L*A*\sigma_{y})$.

### 4.2. Quasi-Static Compression

**Figure 12:**
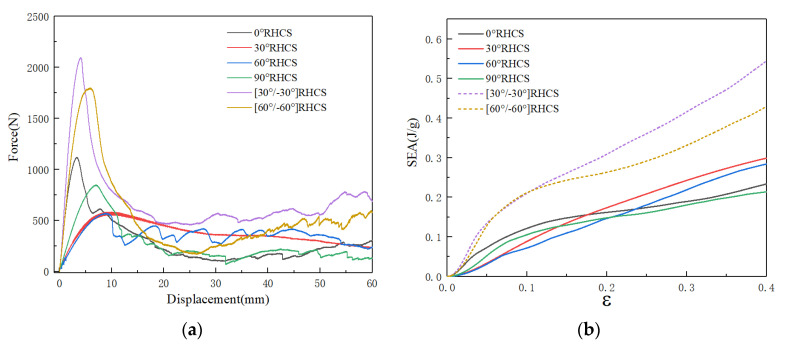
Quasi-static compression: (**a**) the force–displacement curves of RHCSs; (**b**) the SEA curves of RHCSs.

The force–displacement curves and the SEA curves of RHCSs are shown in Figure 12. The structures were fabricated using PLA material, which was useful for studying the deformation modes of RHCSs. The force–displacement curves of RHCSs were divided into two stages: the elastic stage and the plateau stage. Therefore, the pictures that were captured in the elastic and plateau stages were chosen to research the deformation modes.

When 0° re-entrant honeycomb cells were compressed, the inclined wall D_1_E_1_ rotated around the plastic hinge E_1_, and the inclined wall D_1_E_1_ gradually approached the horizon wall E_1_F_1_. With continuous pressure, the cell occurred contracted and showed the negative Poisson’s ratio effect. The deformation process of inclined wall B_1_C_1_ was similar to that of inclined wall D_1_E_1_. When the part connecting inclined wall D_1_E_1_ and plastic hinge E_1_ broke, the force declined rapidly.

The plastic hinge C_2_ of the 30° re-entrant honeycomb cell connected the vertical wall B_2_C_2_ and the vertical wall C_2_F_2_. The plastic hinge D_2_ connected the vertical wall A_2_D_2_ and the vertical wall D_2_E_2_. When the cell was impacted, the vertical wall B_2_C_2_ pushed the inclined wall C_2_F_2_ down and the vertical wall D_2_E_2_ pushed the inclined wall A_2_D_2_ up. Therefore, the cell appeared to extend laterally. A long period of lateral expansion deformation led to the appearance of gentle force displacement curves in the plateau stage. The inclined wall C_2_F_2_ and A_2_D_2_ gradually turned to the horizon with continuous pressure.

**Figure 13:**
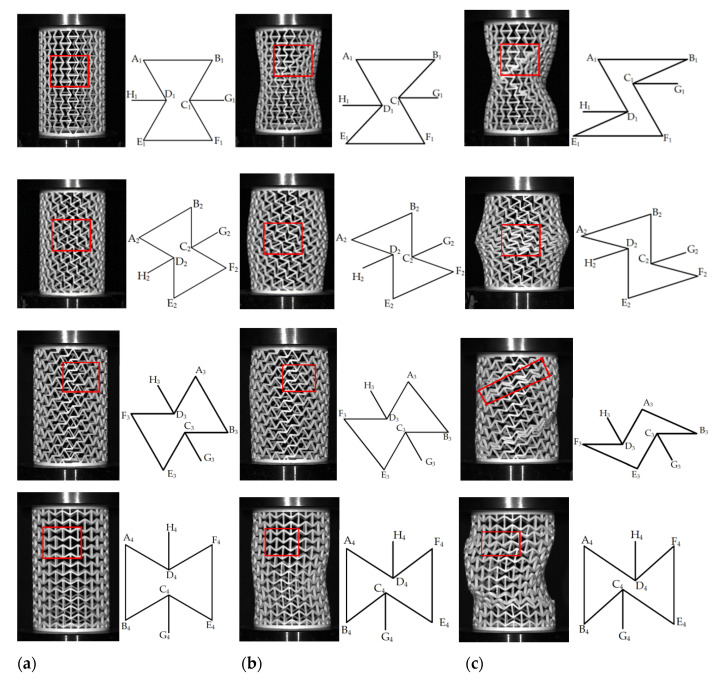
The deformation modes of single-layer RHCSs: (**a**) the initial configuration; (**b**) the deformation modes in the elastic stage; (**c**) the deformation modes in the plateau stage.

As shown in Figure 13c, the deformation modes of 60° re-entrant honeycomb cell passed along with the inclined line, which is marked by red lines. The axial compressive pressure was applied in inclined wall A_3_D_3_, D_3_F_3_, and D_3_H_3_, causing the force on the plastic hinge A_3_ to move was downward. The reaction pressure was applied to the inclined wall C_3_E_3_, B_3_C_3_, and C_3_G_3_, causing upward force on the plastic hinge B_3_. The force on the plastic hinge A_3_ and B_3_ caused the inclined wall A_3_B_3_ to rotate around the plastic hinge B_3_. The deformation modes of 60° RHCS was hierarchically broken; therefore, the force–displacement curve of 60° RHCS was waved.

When the 90° re-entrant honeycomb cell was compressed, the axial compressive pressure was applied to vertical wall D_4_H_4_ to push the plastic hinge D_4_ downward; the reaction pressure was applied to vertical wall C_2_G_2_ to push the plastic hinge C_4_ upward. When the plastic hinge C_4_ and D_4_ approached gradually, the 90° RHCS displayed bulking.

**Figure 14:**
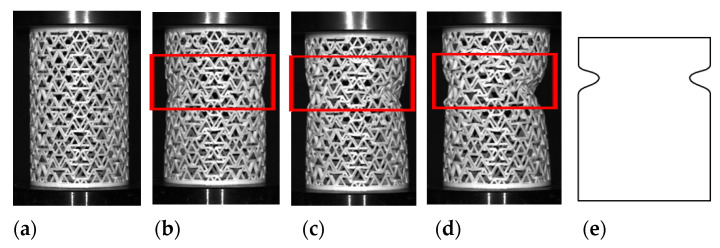
The deformation modes of composite [60°/−60°] RHCS: (**a**) the initial configuration; (**b**) the deformation modes in the elastic stage; (**c**,**d**) the deformation modes in the plateau stage; (**e**) the S deformation shape.

The composite [60°/−60°] RHCS were fabricated by 60° RHCS and −60° RHCS with radial width 1.8 mm, which transformed the composite RHCSs to the isotropic structure. The deformation modes of composite [60°/−60°] RHCS are shown in Figure 14. In the elastic stage, the composite [60°/−60°] RHCS tended to contract in the middle part. In the plateau stage, the middle part displayed bulking with the increasing strain. The deformation shape of composite [60°/−60°] RHCS converted into an S shape.

### 4.3. Low Velocity Impact

The experimental results showed that the PLA material has a brittle fracture property, which is inconvenient for observing the subsequent overall deformation of structures. Therefore, when discussing the energy absorption characteristics of honeycomb cylindrical shells under impact, the aluminum alloy was chosen as the bulk material to eliminate the influence of brittle fracture: Young’s modulus E = 70 GPa, Poisson’s ratio ν = 0.3, material density ρ = 270° kg/m^3^, and yield stress σ_y_ = 130 MPa. To study the energy absorption of RHCSs, we used a low velocity (V) of 10 m/s and a high V of 60 m/s.

**Figure 15:**
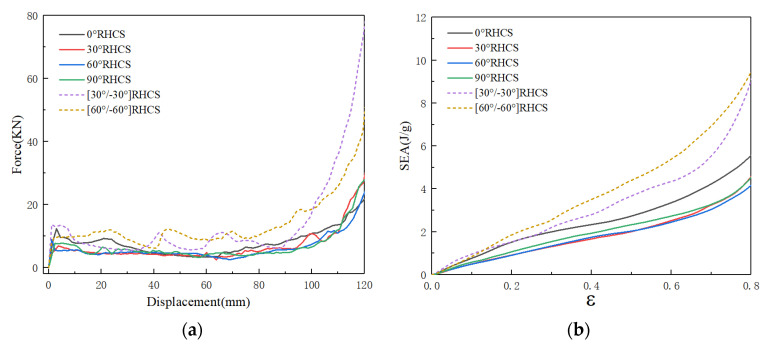
(**a**) The force–displacement curves of RHCSs and (**b**) the SEA curves of RHCSs when V = 10 m/s.

Figure 15 shows force–displacement and the SEA curves of RHCSs with a crushing velocity of 10 m/s. The force–displacement curves of RHCSs were divided into three stages: the elastic stage, the plateau stage, and the dense stage. In the elastic stage, the peak force of composite [30°/−30°] RHCS was the highest. In the plateau stage, the force curves of single-layer RHCSs were close to the others, and the force curves of single-layer RHCSs were lower than the curves of composite RHCSs. In the dense stage, the crushing force of composite RHCSs was much higher than single-layer RHCSs. The SEA curves were closely related to the force–displacement curves, which represent the energy absorption capacity of the structures. The SEA curves of composite RHCSs were higher than those of single-layer RHCSs, which meant that the energy absorption capacity of composite RHCSs is better than that of other RHCSs. In addition, 0° RHCS had the best energy absorption capacity in single-layer RHCSs under low velocity.

**Figure 16:**
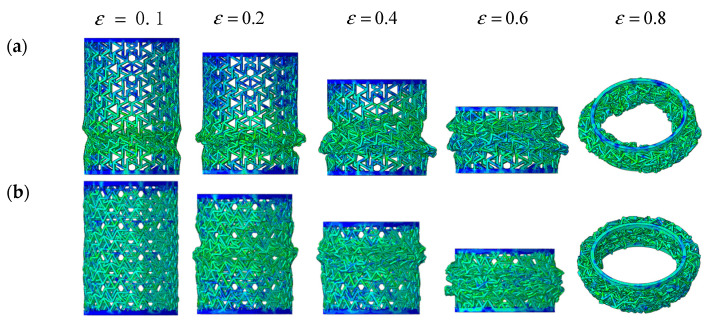
The deformation process of composite RHCSs when V = 10 m/s: (**a**) [30°/−30°] RHCS; (**b**) [60°/−60°] RHCS.

Figure 16 shows the deformation process of composite RHCSs with a crushing velocity of 10 m/s. The force on the composite RHCSs was uniform; therefore, the structures did not appear to expand or shrink. The initial deformation part of [30°/−30°] RHCS occurred in the bottom part, and the S-shape buckling piled up with increasing axial strain, which led to the composite RHCSs being more dense than single-layer RHCSs in the dense stage. This can explain the reason why the energy absorption of composite RHCSs was much higher than that of single-layer RHCSs in the dense stage.

### 4.4. High Velocity Impact

**Figure 17:**
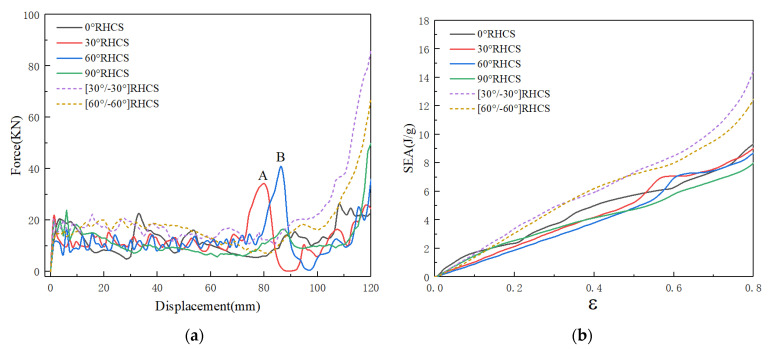
(**a**) The force–displacement curves of RHCSs and (**b**) the SEA curves of RHCSs when V = 60 m/s.

**Figure 18:**
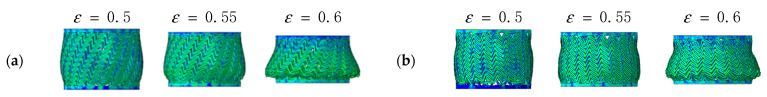
The deformation modes of A and B: (**a**) 30° RHCS; (**b**) 60° RHCS.

**Figure 19:**
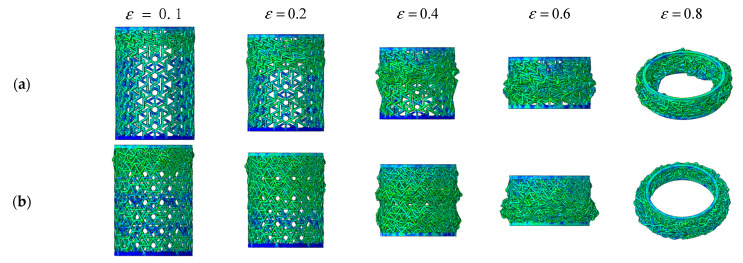
The deformation process of composite RHCSs when V = 60 m/s: (**a**) [30°/−30°] RHCS; (**b**) [60°/−60°] RHCS.

Figure 17 shows force–displacement curves and SEA curves of RHCSs under high velocity. The peak force of 30° and 60° RHCSs was around 80 mm, which is marked in the picture. The force–displacement and SEA curves of composite RHCSs were higher than the single-layer RHCSs under high velocity impact, and it presented better energy absorption than the single-layer RHCSs under high velocity impact. The deformation modes of A and B are shown in Figure 18. When ε = 0.5 and the displacement was 73.5 mm, the porous structures were filled. The structures reached the peak force when ε = 0.55, and the structure was completely compacted and the bottom parts tended to expand. When ε = 0.6 and the displacement was 88.2 mm, the structures were unstable; the bottom parts were broken and radial expansion appeared, which led to a rapid decline in force. The deformation modes of composite RHCSs under high velocity are shown in Figure 19. The initial bulking part of [30°/−30°] and [60°/−60°] RHCSs were in the top part, the bulking section was an S shape, and the S-shaped buckling piled up from the top to the bottom.

**Figure 20:**
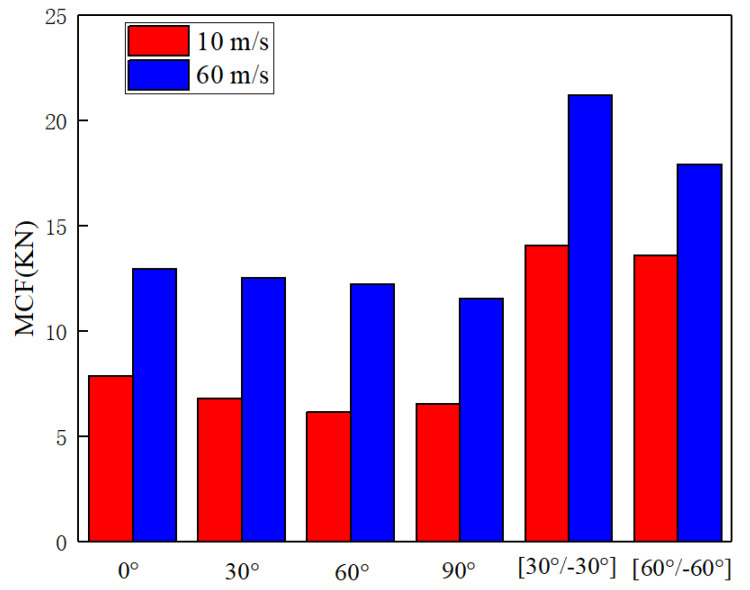
The MCF of RHCSs.

Figure 20 shows the MCF of RHCS. The MCF of composite RHCSs were higher than that of single-layer RHCSs. The non-dimension equivalent plateau stress of RHCSs are listed in Table 2. The non-dimension equivalent plateau stress of composite RHCSs improved significantly compared with that of single-layer RHCSs under low velocity. The non-dimension equivalent plateau stress of [30°/−30°] RHCS improved by 106.8% under low-velocity impact and improved by 68.39% under high-velocity impact compared to the 30° RHCS.

### 4.5. Multi-Layer Composite RHCSs

**Figure 21:**
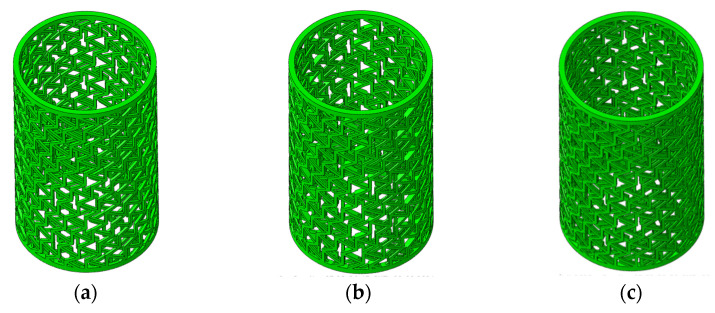
The composite RHCSs: (**a**) double-layer RHCS; (**b**) three-layer RHCS; (**c**) four-layer RHCS.

The composite RHCSs had better energy absorption capacity than the single-layer RHCSs. Therefore, it was necessary to research the influence of multi-layer RHCSs on the energy absorption capacity of composite RHCSs. The multi-layer RHCSs are shown in Figure 21.

**Figure 22:**
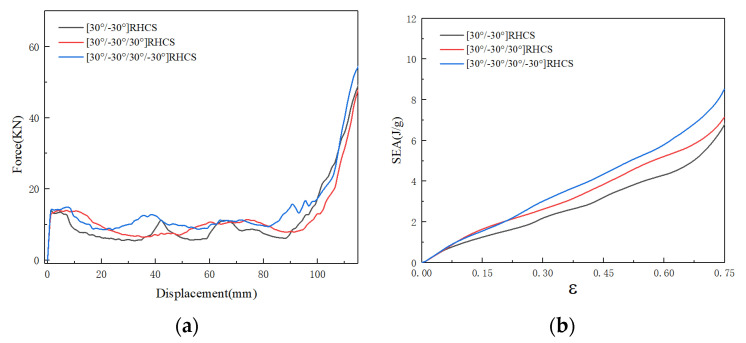
(**a**) The force–displacement curves of multi-layer composite RHCSs and (**b**) the SEA curves of multi-layer composite RHCSs when V = 10 m/s.

The force–displacement curves and SEA curves of multi-layer composite RHCS under low velocity impact are shown in Figure 22. The force of the double-layer composite RHCSs was lower than that of the multi-layer composite RHCSs in most force–displacements, and the SEA curves of the double-layer composite RHCSs were the lowest. This showed that the energy absorption capacity of multi-layer composite RHCSs is better than that of double-layer composite RHCSs at low velocity. In addition, the SEA curves of four-layer composite RHCSs were higher than those of the three-layer composite RHCS. It can be inferred that when the impact velocity was 10 m/s, the energy absorption capacity of multi-layer composite RHCS improved as the number of layers increased.

**Figure 23:**
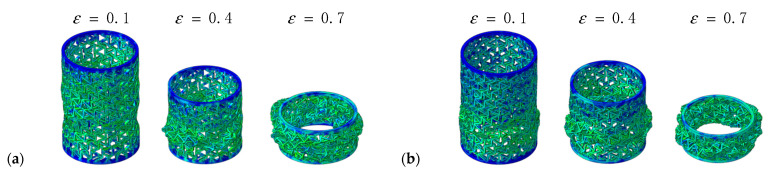
The deformation modes of multi-layer composite RHCSs under low velocity: (**a**) [30°/−30°/30°] RHCS; (**b**) [30°/−30°/30°/−30°] RHCS.

The deformation modes of multi-layer composite RHCSs under low velocity are shown in Figure 23. The initial deformation section appeared in the bottom part, and the deformation shape was ‘S’. With increasing axial strain, the buckling in initial deformation section was piled up from the bottom to the top.

**Figure 24:**
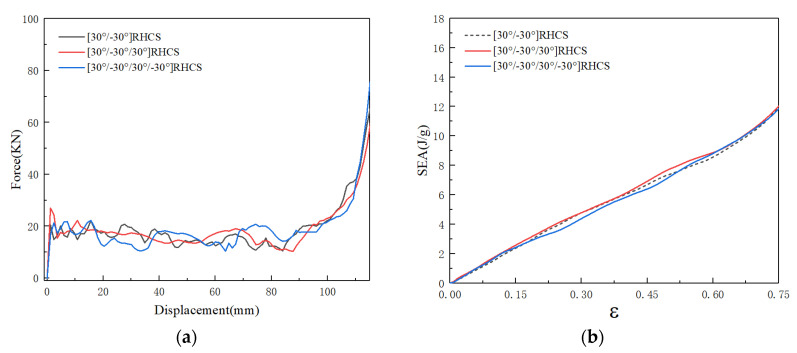
(**a**) The force–displacement curves of multi-layer composite RHCSs and (**b**) the SEA curves of multi-layer composite RHCSs when V = 60 m/s.

**Figure 25:**
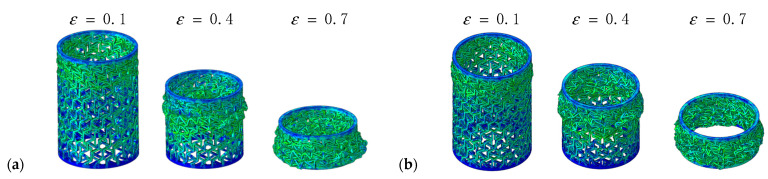
The deformation modes of multi-layer composite RHCSs under high velocity: (**a**) [30°/−30°/30°] RHCS; (**b**) [30°/−30°/30°/−30°] RHCS.

The force–displacement curves and SEA curves of multi-layer composite RHCSs under high-velocity impact are shown in Figure 24. The force–displacement curves and SEA curves are similar to each other. The curves show that the multi-layer composite RHCSs are similar to each other in energy absorption capacity under high velocity. Compared with double-layer composite RHCSs under high velocity, the multi-layer composite RHCSs are not much different in deformation mode. The deformation modes of multi-layer composite RHCSs under high velocity are shown in Figure 25. When the impact velocity was 60 m/s, the deformation modes of multi-layer composite RHCSs and double-layer RHCSs were similar with each other. The deformation from the top to the bottom.

## 5. Conclusions

In this paper, the different orientations of re-entrant honeycomb cylindrical shells (RHCSs) were determined by rotation of the re-entrant honeycomb cell. The deformation modes of the different orientations of single-layer RHCSs were studied by quasi-static compression experiments, and the different orientations of single-layer RHCSs did not show the negative Poisson’s ratio effect. The energy absorption capacity of different orientations of single-layers RHCSs were similar to the conventional RHCSs, measured using experimental and simulation methods.

In addition, the composite RHCSs were constructed by connection of the different orientations of single-layers RHCSs. The double-layer composite RHCSs had the same size as the single-layer RHCSs. However, the double-layer composite RHCSs had a better energy absorption capacity than single-layer RHCSs, measured using experimental and simulation methods. Furthermore, when the composite RHCSs were impacted by low velocity, the energy absorption capacity of composite RHCSs improved as the number of layers increased.

## Figures and Tables

**Table 1:** The geometrical dimensions of RHCSs.

Specimen	Radius (mm)	Radial Width (mm)	Axial Height (mm)	Relative Densities
0° RHCS	44.56	3.6	146.56	0.405
30° RHCS	44.1	3.6	146.56	0.405
60° RHCS	44.56	3.6	146.56	0.405
90° RHCS	44.1	3.6	146.56	0.405
[30°/−30°] RHCS	44.1	3.6	146.56	0.405
[60°/−60°] RHCS	44.56	3.6	146.56	0.405
[30°/−30°/30°] RHCS	44.1	3.6	146.56	0.405
[30°/−30°/30°/−30°] RHCS	44.1	3.6	146.56	0.405

**Table 2:** The non-dimension equivalent plateau stress of RHCSs.

Specimen	When V = 10 m/s, the Non-Dimension Equivalent Plateau Stress (MPa)	Improvement (Compared with 30° RHCS)	When V = 60 m/s, the Non-Dimension Equivalent Plateau Stress (MPa)	Improvement (Compared with 30° RHCS)
0° RHCS	0.0602	14.2%	0.0989	2.17%
30° RHCS	0.0527	0	0.0968	0
60° RHCS	0.0472	−10.4%	0.0934	−3.51%
90° RHCS	0.0471	−10.6%	0.0891	−7.95%
[30°/−30°] RHCS	0.109	106.8%	0.163	68.39%
[60°/−60°] RHCS	0.104	97.3%	0.137	41.53%

**Figure A1:**
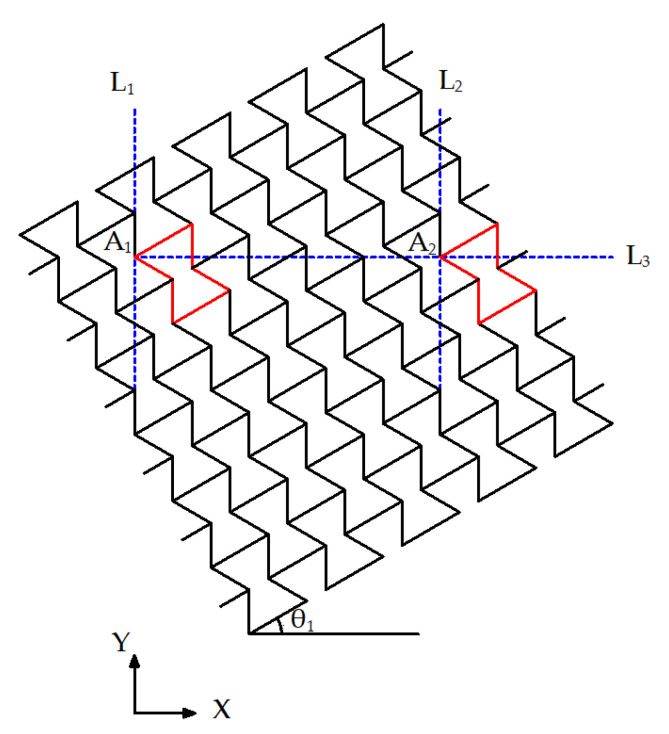
*θ*_1_ re-entrant honeycomb layer.

**Figure A2:**
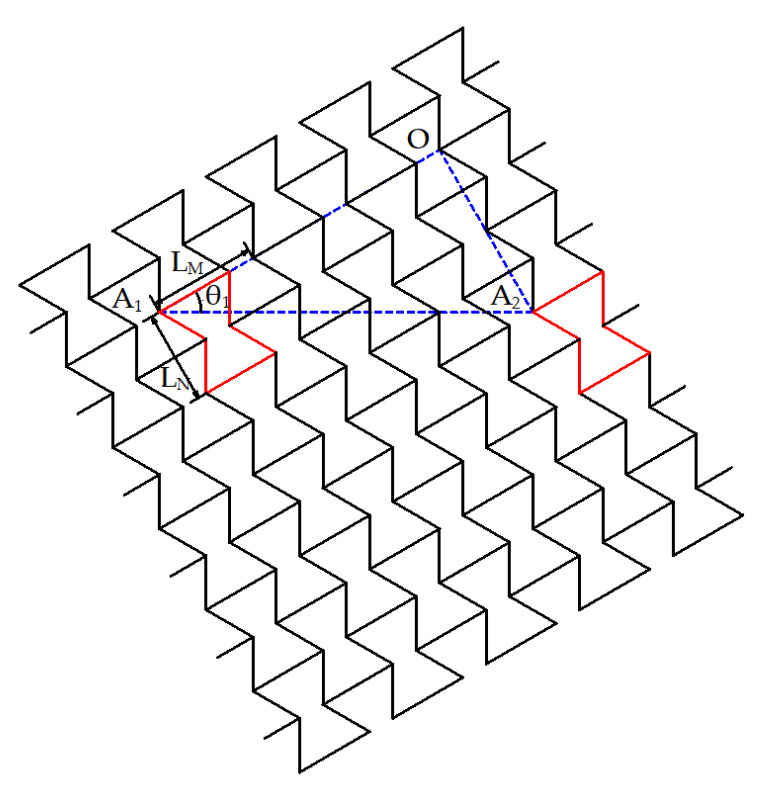
The geometrical configuration between point A_1_ and point A_2_.

**Figure A3:**
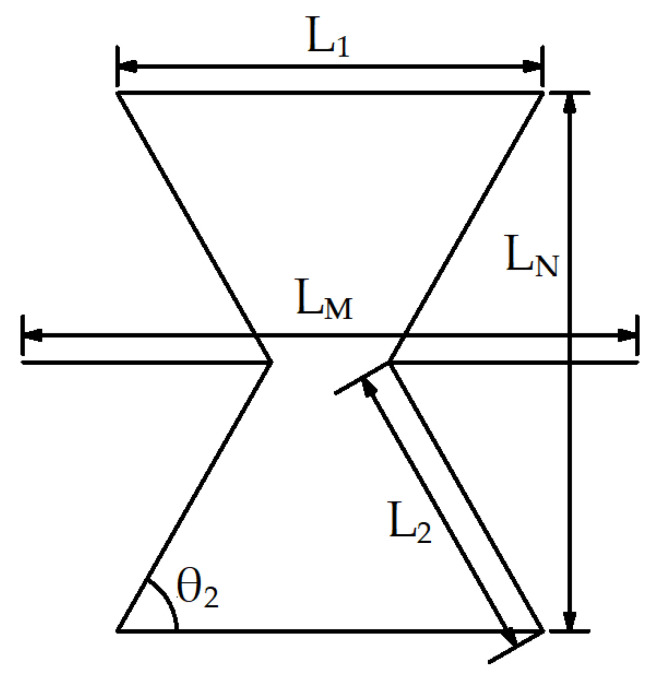
Geometrical configuration of a re-entrant honeycomb cell.

## Data Availability

Data is contained within the article.

## Appendix A

To ensure the stability of the structure and avoid structural defects, the honeycomb cylindrical shells need to be joined to form a complete cell. Points A_1_ and A_2_ on the horizontal line L_3_, as well as points A_1_ and A_2_ in the same position of re-entrant honeycomb cell are depicted in Figure A1. By drawing vertical lines L_1_ and L_2_ through point A_1_ and point A_2_, the right section of vertical lines L_1_ and the left section of vertical lines L_2_ can form complete honeycomb cylindrical shells. Point A_1_ and point A_2_ are called coincident points. The geometrical configuration between point A_1_ and point A_2_ are shown in Figure A2. The red lines represent the re-entrant honeycomb unit which were analysed, and the blue lines represent horizontal and vertical lines.

Therefore, the distance between point A_1_ and point O is m times the width of the re-entrant honeycomb cell. The distance between point A_2_ and point O is n times the width of the re-entrant honeycomb cell, which can be expressed as:

(A1) $$\tan\theta_{1}=\frac{n\cdot L_{N}}{m\cdot L_{M}}(m=1,2,3,\ldots \ldots ;n=1,2,3,\ldots \ldots )$$

Figure A3 shows the geometrical configuration of a re-entrant honeycomb cell; *L_N_* and *L_M_* can be expressed as:

(A2) $$L_{N}=2L_{2}\sin\theta_{2}$$

(A3) $$L_{M}=2(L_{1}-L_{2}\cos\theta_{2})$$

Substituting (A2) and (A3) into (A1) provides:

(A4) $$\begin{array}{ll} \tan\theta_{1} & =\frac{n\cdot L_{N}}{m\cdot L_{M}}=\frac{n}{m}\cdot \frac{2L_{2}\sin\theta_{2}}{2(L_{1}-L_{2}\cos\theta_{2})}\\ & =\frac{n}{m}\cdot \frac{L_{2}\sin\theta_{2}}{\left(L_{1}-L_{2}\cos\theta_{2}\right)}(m=1,2,3,\ldots \ldots ;n=1,2,3,\ldots \ldots ) \end{array}$$

To find a real number k to make $L_{1}=k\cdot L_{2}\cos\theta_{2}$, the expression (A4) can be expressed as:

(A5) $$\begin{array}{ll} \tan\theta_{1} & =\frac{n}{m}\cdot \frac{L_{2}\sin\theta_{2}}{\left(k\cdot L_{2}\cos\theta_{2}\;-\;L_{2}\cos\theta_{2}\right)}=\frac{n}{m}\cdot \frac{L_{2}\sin\theta_{2}}{(k-1)\cdot L_{2}\cos\theta_{2}}=\frac{n}{m}\cdot \frac{1}{\left(k-1\right)}\frac{L_{2}\sin\theta_{2}}{L_{2}\cos\theta_{2}}\\ & =\frac{n}{m}\cdot \frac{1}{\left(k-1\right)}\tan\theta_{2}(m=1,2,3,\ldots \ldots ;n=1,2,3,\ldots \ldots ) \end{array}$$

In this paper, the experiments mainly focused on the different orientations of honeycomb cylindrical shells. The geometrical configuration of re-entrant honeycomb cell did not change, which means that $\theta_{2}$ and k did not change when $\theta_{1}$ changed.

When $\theta_{2}=60^{{}^{\circ}}$, k=3, n=2, m=3, $\theta_{1}=30^{{}^{\circ}}$;

When $\theta_{2}=60^{{}^{\circ}}$, k=3, n=2, m=1, $\theta_{1}=60^{{}^{\circ}}$.
